# Abrine Elicits Liver Carcinoma Immunity and Enhances Antitumor Efficacy of Immune Checkpoint Blockade by Modulating PD-L1 Signaling

**DOI:** 10.1155/2022/7609676

**Published:** 2022-05-12

**Authors:** Shaowu Zhang

**Affiliations:** Department of Rehabilitation Medicine, Hanyang Hospital Affiliated to Wuhan University of Science and Technology, Wuhan, Hubei Province, China

## Abstract

**Background:**

Liver cancer is the most malignant type of human malignancies. In recent years, immune therapy that targets the immune check points such as programmed cell death ligand 1 (PD-L1) has achieve great success. Abrine is the dominant alkaloid in *Abrus cantoniensis* and *Abrus precatorius* Linn. that exhibited anticancer effect. This work is aimed at studying the effects of abrine in immunity of liver cancer.

**Methods:**

Cell viability, proliferation, and migration were assessed by CCK-8, Edu, and Transwell assay. Cell apoptosis was checked by flow cytometry. Tumor growth was determined by an *in vivo* xenograft model. Quantitative real-time PCR assay was conducted to evaluate the levels of KAT5 and PD-L1. T cells and liver cancer cells were cocultured in a Transwell system, and the levels of PD-L1 and PD-1 was checked by flow cytometry. The interaction between KAT5 and PD-L1 was determined by ChIP assay.

**Results:**

Abrine treatment suppressed liver tumor growth both in vitro and in vivo and simultaneously decreased the level of PD-L1 and KAT5. In the coculture system, treatment with abrine inhibited proliferation and activity of cocultured T cell. KAT5 epigenetically elevated recruitment of H3k27ac and RNA polymerase II to PD-L1 promoter region. Ectopic expression of KAT5 and PD-L1 reversed the function of abrine on tumor growth and T cell function.

**Conclusion:**

Abrine modulated growth and apoptosis of liver cancer cells and regulated proliferation and activation of T cells through the KAT5/PD-L1 axis.

## 1. Introduction

Liver cancer is the one of the most malignant cancer type globally and the second leading cause of cancer-related death [1]. The primary liver cancers cholangiocarcinoma and hepatocellular carcinoma (HCC) account for over 75% [1]. Liver cancers are mostly diagnosed at late stage and present a 5-year overall survival less than 10%, owing to unobvious symptoms and aggressive phenotype [2, 3]. The main pathological factors of liver cancer include nonalcoholic fatty liver disease (NAFLD), alcohol, cirrhosis, and virus infection such as hepatitis B virus (HBV) and hepatitis C virus (HCV) [4].

Current therapeutic manners for liver cancer mainly include surgical resection, liver transplant, and the multikinase inhibitor sorafenib; nevertheless, the low-ratio eligibility for surgery and high recurrence rates largely limited the prognosis of patients [5]. Over the past decade, immune therapy has become a prevalent therapeutic strategy in multiple cancer types [6]. Among the immune therapies, the blockade of immune checkpoints, such as PD-L1 and CTLA-4, is the most effective approaches [7]. Studies have indicated elevated PD-L1 level in liver cancer cells, and its correlation with metastasis and poorer prognosis of patients [8, 9]. Moreover, high level of PD-L1 on cancer cells induced abnormal activation and function of T cells [10, 11].

Histone acetylation is a reversible posttranslational modification that catalyzed by lysine acetyltransferases (KATs) and is critical for regulation of gene expression [12]. KAT5, also known as Tip60, is an important member of histone acetyltransferases (HATs) and participates in various cellular processes, such as cell proliferation, migration, and the DNA damage response and repair [13, 14]. KAT5 modulates the stabilization and function of c-Myc, which further affects the progression of breast cancer and thyroid cancer [15–17].

Traditional Chinese medicine has a long history in treatment of disease, including cancers [12]. Abrine is the dominant alkaloid extracted from *Abrus cantoniensis* and *Abrus precatorius* Linn. [18]. It has been reported that abrine inhibited the activity of indoleamine 2,3-dioxygenase 1 (IDO1), the important immunoregulatory enzyme, and suppressed tumor growth [18]. Abrine also exhibits antioxidative function, which suggested its anti-inflammatory effect [19]. Increasing evidences have revealed the targets of abrine, such as peroxisome proliferator-activated receptors (PPARs) and isocitrate dehydrogenase (IDH), which supported the application of abrine in disease treatment [20, 21].

In this work, we evaluated the function of abrine on immune response of liver cancer, determined that abrine inhibited liver cancer cell growth, suppressed PD-L1 expression, and activated T cell proliferation and activation. Mechanistically, KAT5 epigenetically elevated acetylation and transcription of PD-L1 promoter. Our findings disclosed novel regulatory mechanisms of abrine for liver cancer treatment.

## 2. Materials and Methods

### 2.1. Cell Lines and Treatment

Human liver cancer cell lines HepG2 and Huh7 were obtained from American Type Culture Collection (ATCC, USA), maintained in DMEM (Thermo, USA) medium complemented with 10% fetal bovine serum (FBS; Hyclone, USA) and 1% penicillin/streptomycin (Sigma, China). Human peripheral blood mononuclear cells (PBMCs) and T cells were cultured in RPMI-1640 medium with 10% FBS and 1% penicillin/streptomycin. All cells were maintained in 37°C incubator that filled with 5% CO_2_. The coculture system consists of cancer cells and T cells in lower and upper chambers of Transwell, at a ratio of 1 : 10. The culture medium of cancer cells were collected after 24 hours incubation, centrifuged at 13000 rpm for 10 minutes, and then, mixed with equal amount of fresh medium to obtain conditioned medium. Abrine (20 *μ*M) [22] and/or 200 ng/mL IFN-*γ* (Sigma, USA) was added to stimulate cancer cells for 24 hours.

### 2.2. Cell Transfection

KAT5-overexpressing vectors (KAT5 OE), PD-L1-overexpressing vectors (PD-L1 OE), KAT5 siRNAs (siKAT5-1 and siKAT5-2), and negative control (NC) were designed and synthesized by Gene Pharma (China). For cell transfection, Lipofectamine 2000 (Invitrogen, USA) was used for as per the manufacturer's protocol.

### 2.3. Cell Viability and Apoptosis

Cell viability was assessed by cell counting kit 8 (CCK-8; Beyotime, China) and Edu assay. For CCK-8 assay, cells were seeded into 96-well plates and incubated for 24 hours. Then, 20 *μ*L CCK-8 reagent was added to incubate for two hours. Absorbance values at 450 nm were measured by a microplate reader (Thermo, USA). The Edu assay was performed in line with manufacturer's instruction. In short, cells were stained with Edu reagent at room temperature for one hour, fixed with formaldehyde, and permeabilized in 0.5% Triton X-100. The nuclei were labeled with Hoechst 33342 (Sigma, USA). Images were taken by confocal microscope (Leica, Germany). Cell apoptosis was determined by Annexin V/PI Apoptosis Detection kit (Thermo, USA) following manufacturer's protocol.

### 2.4. Transwell Assay

HepG2 and Huh7 cells in FBS-free DMEM medium were placed into the upper chamber of a 24-well Transwell plate, and the lower chamber contained media with 10% FBS. After incubation for 48 hours, the cells on the lower side of the chamber were fixed, stained with crystal violet. Five fields of view were randomly selected to observe the cells under a 400-fold microscope, and the cells were photographed and counted.

### 2.5. Quantitative Real-Time PCR (qRT-PCR)

Cells were homogenized by ice-cold Trizol reagent (Thermo, USA) to obtain total RNA. Then, 1 *μ*g RNA was synthesized to cDNA by using first-strand cDNA synthesis kit (Thermo, USA). Gene expression was measured by SYBR Green/Rox Mixture (Thermo, USA) with 2^−ΔΔCt^ method and normalized to GAPDH level. Reaction conditions: 95°C for 2 min; 40 cycles of 95°C for 10 s, 55°C for 15 s, 72°C for 10 s; 95°C for 1 min, 60°C for 30 s, and 95°C for 30 s. Reaction system: SYBN Premix Ex Tag solution 10 *μ*L, upstream primer (20 *μ*mol/L) 0.2 *μ*L, downstream primer (20 *μ*mol/L) 0.2 *μ*L, ddH2O 7.6 *μ*L, cDNA 2 *μ*L, and total 20 *μ*L. PD-L1 upstream primer: 5′-CTGTTTTTCAA TCTCCGGGTA-3′, downstream primer: 5′-TGAAAGCAG TGTTCAGGGTCT-3′, KAT5 upstream primer: 5′-CGACCACTTTGTCAAGCTCA-3′, downstream primer: 5′-AGGGGTCTACATGGCAACTG-3′, *β*-actin is an internal reference, upstream primer: 5′-GCCACTGCCGCATCCTCTTC-3′, downstream primer: 5′-AGCCTCAGGGCATCGGAACC-3′.

### 2.6. T Cell Priming

The T cells were obtained from PBMCs by using a Pan-T cell isolation kit (Miltenyi Biotec, Germany) in accordance with the manufacturer's description. The activation of immune cells was conducted by using a T Cell Activation/Expansion Kit (Miltenyi Biotec, Germany). In short, MACSiBead was labeled with CD28, CD2, and CD3 antibody, then used to incubate with PBMCs or T cells.

### 2.7. Chromatin Immunoprecipitation (ChIP) Assay

Liver cancer cells were homogenized to obtain genome, followed by sonication. The antibodies against KAT5 (Abcam, USA), H3k27ac (Abcam, USA), and RNA polymerase II (Abcam, USA) were then added into the genome fragments to incubate at 4°C overnight. The samples were then incubated with Protein A agarose at 4°C for 6 hours, washed with PBS, then eluted to obtain DNA. Then, qRT-PCR was conducted to evaluate DNA binding.

### 2.8. Flow Cytometry

For detection of surface biomarkers PD-1, PD-L1, CD4, and CD8, cells were collected and labeled with corresponding antibodies in dark for 2 hours, then subjected to FACSCanto II flow cytometry (BD Biosciences, USA).

### 2.9. Xenograft Tumor Model

BALB/c nude mice were purchased from Vital River Laboratory (China). HepG2 cells (5 × 10^5^) suspended in 100 *μ*L PBS were inoculated into the fat pad of BALB/c mice. When the tumor volume reached 100 mm^3^, mice were intraperitoneally injected with PD-L1 antibody (10 mg/kg body weight). And abrine (15 mg/kg body weight) were administrated through tail vein injection. Twenty days later, the mice were sacrificed by cervical dislocation, and the liver tumor tissue of the mice was dissected and weighed, and the weight and volume of the tumor tissue were calculated. Tumors were then fixed in 4% PFA, embedded with paraffin, and made into 4 *μ*m thick slices. The proliferative biomarker KI-67 was evaluated by incubation with anti-Ki67 antibody (1 : 100, Abcam, USA) overnight at 4°C and visualized by DAB staining (Beyotime, China). All animal procedures were performed following the Guidelines of Animal Care and Use of Hanyang Hospital Affiliated to Wuhan University of Science and Technology, no. HY29372.

### 2.10. Statistics

Data were shown as mean ± S.D. Student's *t*-test was used for comparison between two groups. And one-way ANOVA was adopted for comparison between multiple groups. *P* < 0.05 were considered statistically significant. The statistical analysis was conducted by using the SPSS software (Version 19.0).

## 3. Results

### 3.1. Abrine Suppresses Liver Tumor Growth

**Figure 1:**
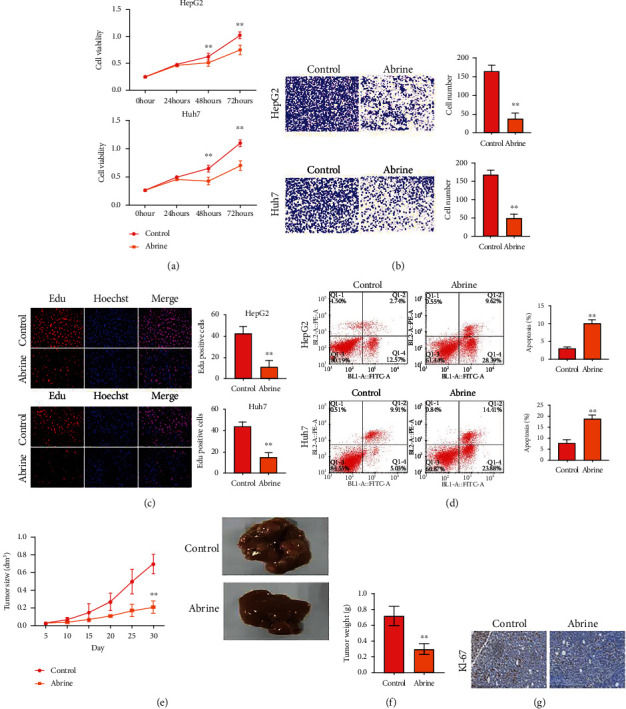
Abrine suppresses liver tumor growth. (a) Cell viability was checked by CCK-8 assay. (b) Cell migration measured by Transwell assay. (c) Cell proliferation was assessed by Edu assay. (d) Cell apoptosis evaluated by flow cytometry. (e–g) Xenograft tumor model was established using HepG2 cells. Then, tumor growth curve (e) and tumor weight (f) were recorded. The expression of KI-67 was assessed by IHC staining. ^∗∗^*P* < 0.01 vs. control.

The cell viability was detected by CCK-8, and it was found that the viability of HepG2 and Huh7 cells of liver cancer was significantly decreased after abrin treatment (*P* < 0.05) (Figure 1(a)). The cell migration ability was detected by Transwell assay, and it was found that the migration ability of liver cancer HepG2 and Huh7 cells was significantly inhibited after abrin treatment (*P* < 0.05), Figure 1(b). The cell proliferation ability was detected by Edu assay, and it was found that the proliferation ability of hepatoma HepG2 and Huh7 cells was significantly inhibited after treatment with acacia (*P* < 0.05) (Figure 1(c)). Flow cytometry detection of apoptosis ability found that the apoptosis of liver cancer HepG2 and Huh7 cells was significantly increased after treatment with acacia (*P* < 0.05) (Figure 1(d)). Based on the above results, it was found that acacia had stronger antitumor effect on HepG2 cells, so HepG2 was selected for subsequent experiments. A xenograft tumor model was established with HepG2 cells, and it was found that acacia can significantly inhibit tumor growth (Figures 1(e) and 1(f)). The expression of KI-67 in liver tissue was detected by IHC method, and it was found that the expression level of tumor proliferation biomarker KI-67 in the liver tissue of mice treated with acacia was significantly decreased (*P* < 0.05) (Figure 1(g)).

### 3.2. Abrine Impairs Expression of PD-L1 and Activation of T Cells

**Figure 2:**
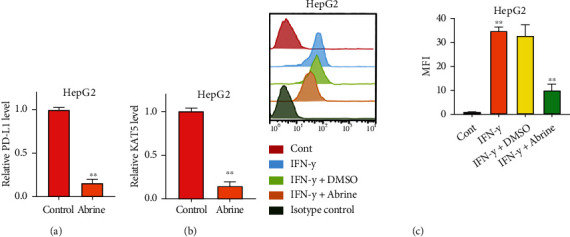
Abrine impairs expression of PD-L1. (a, b) RNA levels of PD-L1 and KAT5 in cancer cells under treatment of abrine were checked by qRT-PCR. (c) Liver cancer cells were stimulated with IFN-*γ* and treated with or without abrine, and PD-L1 expression was detected by flow cytometry. ^∗∗^*P* < 0.01 vs. control.

**Figure 3:**
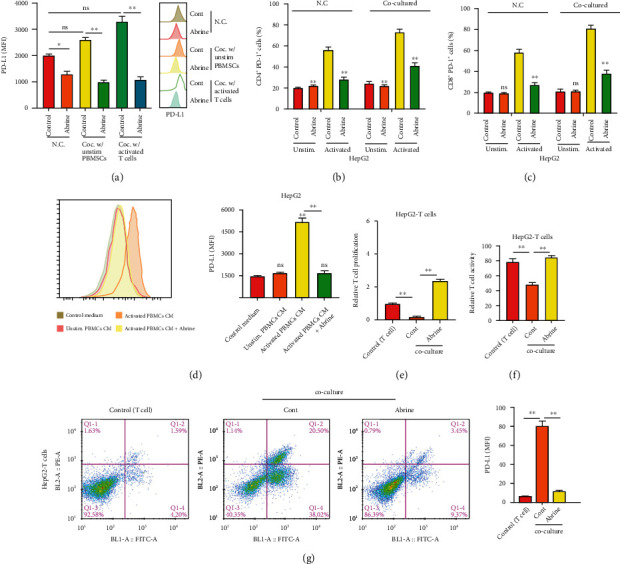
Abrine stimulates activation and function of T cells. (a–c) The unstimulated or activated PBMCs were set in top chambers of the Transwell; the liver cancer cells were placed in the bottom chambers. The level of PD-L1 in HepG2 cells (a) and percentage of CD4+/PD-1+ T cells (b) and CD8+/PD-1+ T cells (c) were checked by flow cytometry. (d) The PD-L1 level of HepG2 cells under treatment with conditioned medium of PBMCs detected by flow cytometry. (e) T cell proliferation under coculture with HepG2 and Huh7 cells was determined by CCK-8 assay. (f) T cell activity under con-culture with HepG2 and Huh7 cells was measured by flow cytometry detection of CD8+ T cells. (g) Apoptosis of T cells were determined by flow cytometry. ^∗∗^*P* < 0.01 vs. control.

We also found a notable declination of PD-L1 under abrine treatment, along with decreased level of acetyltransferase KAT5 (Figures 2(a) and 2(b)). Moreover, abrine treatment abolished the PD-L1 expression caused by IFN-*γ* stimulation in liver cancer cells (Figure 2(c)). We next established a Transwell coculture system to test the effects of abrine on crosstalk between cancer cells and T cells. As shown in Figure 3(a), under coculture condition, PD-L1 level of cancer cells were suppressed. Moreover, the portion of activated T cells, including the CD4+/PD-1+ T cells (Figure 3(b)) and CD8+/PD-1+ T cells (Figure 3(c)), was decreased by coculture with abrine-treated HepG2 cells, comparing with that under treatment of DMSO. To confirm that T cells affect liver cancer cells function through secreting factors, we collected the culture medium from PBMCs treated with abrine and treated the liver cancer cells. We found that the conditioned medium from activated PBMCs elevated the PD-L1 expression, and abrine treatment reversed this effect (Figure 3(d)). On the other hand, coculture with liver cancer cells notably suppressed the proliferation (Figure 3(e)) and activity (Figure 3(f)) of T cells. Consistently, the treatment with abrine remarkably decreased the apoptosis of T cells (Figure 3(g)).

### 3.3. Abrine Epigenetically Regulates PD-L1 Expression via Recruiting KAT5

**Figure 4:**
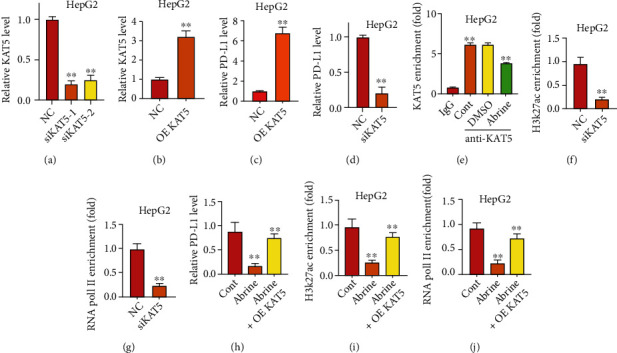
Abrine epigenetically regulates PD-L1 expression via recruiting KAT5. (a–d) Levels of KAT5 (a, b) and PD-L1 (c, d) after transfection with siKAT5 and KAT5 overexpressing vectors (OE KAT5) were detected by qRT-PCR assay. (e) ChIP assay to check the interaction of KAT5 to PD-L1 promoter region. (f, g) Enrichment of H3K27Ac (f) and RNA pol II (g) on the PD-L1 promoter was assessed by ChIP assay under transfection with siKAT5 or NC. (h) Relative level of PD-L1 after transfection with KAT5 overexpressing vector (OE KAT5). Enrichment of H3K27Ac (i) and RNA pol II (j) on the PD-L1 promoter was assessed by ChIP assay under treatment with abrine and transfection with KAT5 overexpressing vector (OE KAT5). ^∗∗^*P* < 0.01 vs. control and abrine group.

We observed a decrease of KAT5 along with the PD-L1 in abrine-treated liver cancer cells; here, we wonder if KAT5 mediates the regulation of PD-L1. We performed overexpression and knockdown of KAT5 with specific siRNAs and overexpressing vectors (Figures 4(a) and 4(b)), the results showed that compared with the NC group, the relative expression levels of siKAT5 in the siKAT5-1 group and siKAT5-2 group were significantly decreased (*P* < 0.05), and the relative expression level in the OE-KAT5 group was significantly increased (*P* < 0.05). The above indicated that the KAT5 overexpression and knockdown model was successfully constructed. We chose siKAT5-1 for following experiments. Noteworthy, we determined that the level of PD-L1 elevated and declined following overexpression and depletion of KAT5 (Figures 4(c) and 4(d)). Results from ChIP assay indicated that KAT5 directly interacted with the promoter region of PD-L1; abrine treatment directly impaired this interaction (Figure 4(e)). Besides, knockdown of siKAT5 notably decreased the enrichment of H3k27Ac and RNA polymerase II on PD-L1 gene, compared with that of the NC group (Figures 4(f) and 4(g)). Further study demonstrated that KAT5 overexpression reversed the decreased level of PD-L1 caused by abrine treatment (Figure 4(h)) and simultaneously upregulated the enrichment of H3k27Ac and RNA polymerase II (Figures 4(i) and 4(j)).

### 3.4. KAT5 Knockdown Affects Liver Cancer Cell Growth and T Cell Function

**Figure 5:**
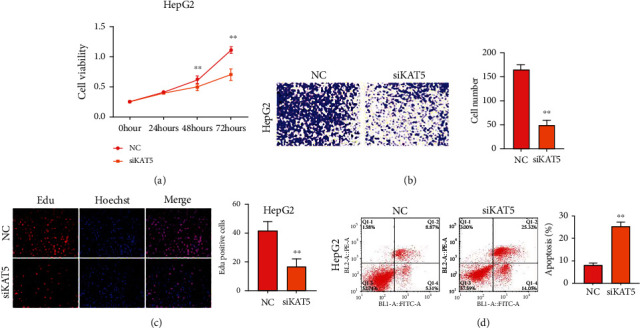
KAT5 depletion inhibits growth of liver cancer cells. HepG2 cells was transfected with siKAT5 or NC. The viability (a), migration (b), proliferation (c), and apoptosis (d) were measured by CCK-8, Transwell assay, and flow cytometry, respectively. ^∗∗^*P* < 0.01 vs. control.

**Figure 6:**
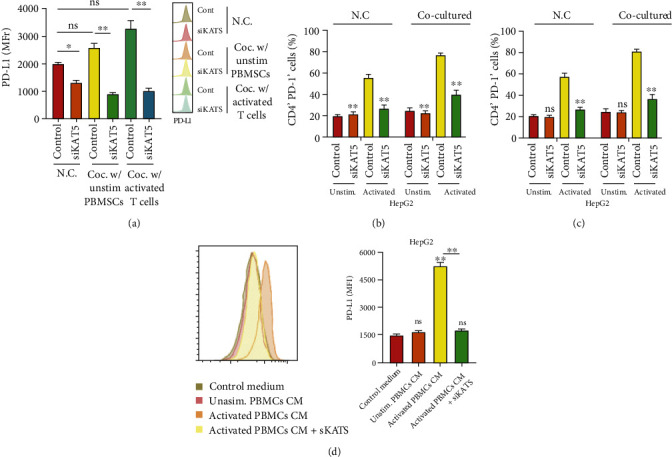
KAT5 knockdown suppresses T cell function. (a–c) The unstimulated or activated PBMCs were set on top chambers of the Transwell; the liver cancer cells depleted of KAT5 were placed in the bottom chambers. The level of PD-L1 in HepG2 cells (a) were checked by flow cytometry. The percentages of CD4+/PD-1+ T cells (b) and CD8+/PD-1+ T cells (c) were checked by flow cytometry. (d) The PD-L1 level of HepG2 cells under treatment with conditioned medium of PBMCs detected by flow cytometry.

Depletion of KAT5 caused notable inhibition of cell viability and migration as determined by CCK-8 (Figure 5(a)), Transwell assay (Figure 5(b)), and Edu assay (Figure 5(c)). Results from flow cytometry suggested that siKAT5 significantly increased the apoptosis of liver cancer cells (Figure 5(d)). We also determined the function of KAT5 in crosstalk between T cells and liver cancer cells. As shown in Figure 6(a), siKAT5-transfected liver cancer cells decreased the expression of PD-L1 under stimulated and unstimulated condition. The portion of CD4+/PD-1+ T cells (Figure 6(b)) and CD8+/PD-1+ T cells (Figure 6(c)) were decreased by coculture with KAT5-depleted HepG2 cells. Treatment with the conditioned medium from activated PBMCs stimulated PD-L1 expression of liver cancer cells, while KAT5 knockdown reversed this effect (Figure 6(d)).

### 3.5. Abrine Stimulates Proliferation and Activation of T Cells through Regulating KAT5/PD-L1 Axis

**Figure 7:**
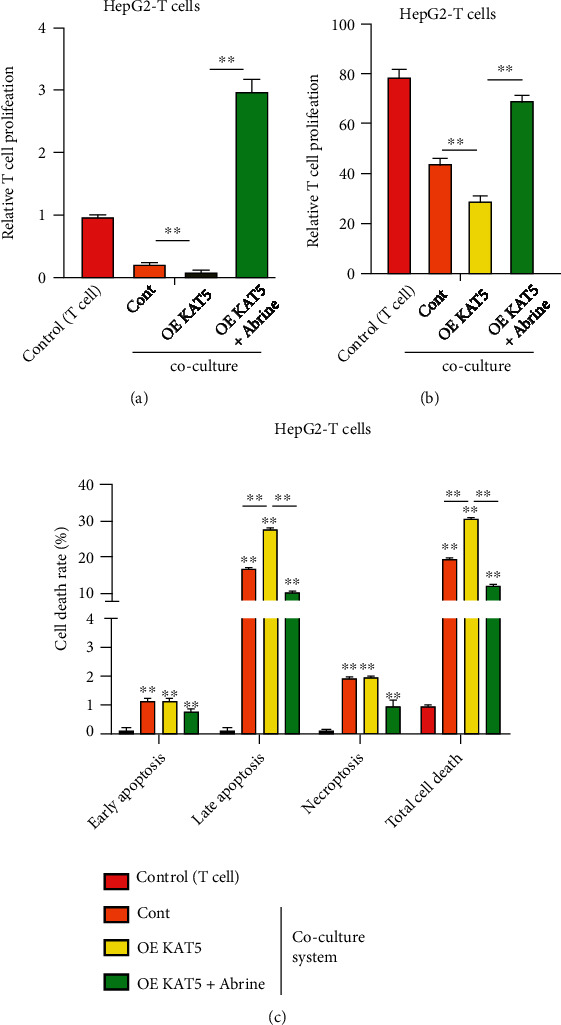
Abrine stimulates activation of T cells via KAT5. HepG2 cells were transfected with KAT5 overexpressing vectors and cultured with T cells with or without abrine treatment. (a) T cell proliferation was determined by CCK-8 assay. (b) T cell activity was measured by flow cytometry detection of CD8+ T cells. (c) Apoptosis of T cells were determined by flow cytometry. ^∗∗^*P* < 0.01 vs. control.

**Figure 8:**
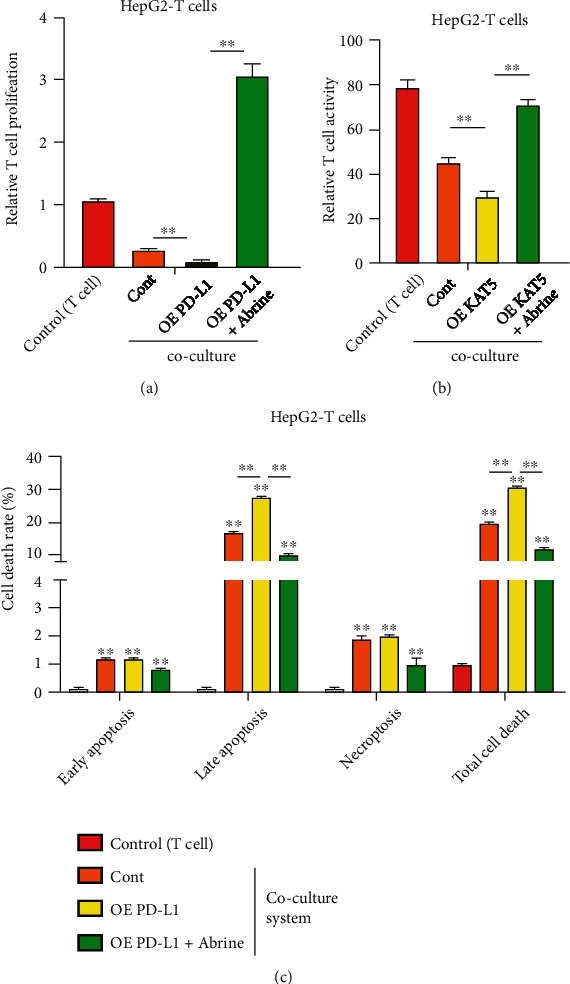
Abrine stimulates activation of T cells via PD-L1. HepG2 cells were transfected with PD-L1 overexpressing vectors, and cultured with T cells with or without abrine treatment. (a) T cell proliferation was determined by CCK-8 assay. (b) T cell activity was measured by flow cytometry detection of CD8+ T cells. (c) Apoptosis of T cells were determined by flow cytometry. ^∗∗^*P* < 0.01 vs. control.

To explore whether KAT5 mediates abrine-suppressed liver tumor growth through regulating T cells function, we evaluated proliferation and activation of T cells that cocultured with liver cancer cells under abrine treatment and KAT5 overexpression. The proliferation and activity of T cells that cocultured with KAT5-overexpressed liver cancer cells were notably suppressed comparing with those cocultured with control cancer cells, whereas treatment with abrine significantly stimulated T cell proliferation and activity (Figures 7(a) and 7(b)). Consistently, the treatment with abrine remarkably decreased KAT5-induced apoptosis of T cells in the coculture system (Figure 7(c)). Moreover, abrine also reversed PD-L1 overexpression-suppressed proliferation and activation of T cells (Figures 8(a) and 8(b)), along with elevated T cells apoptosis (Figure 8(c)).

### 3.6. Abrine Inhibits Liver Tumor Growth through Regulating KAT5/PD-L1 Axis

**Figure 9:**
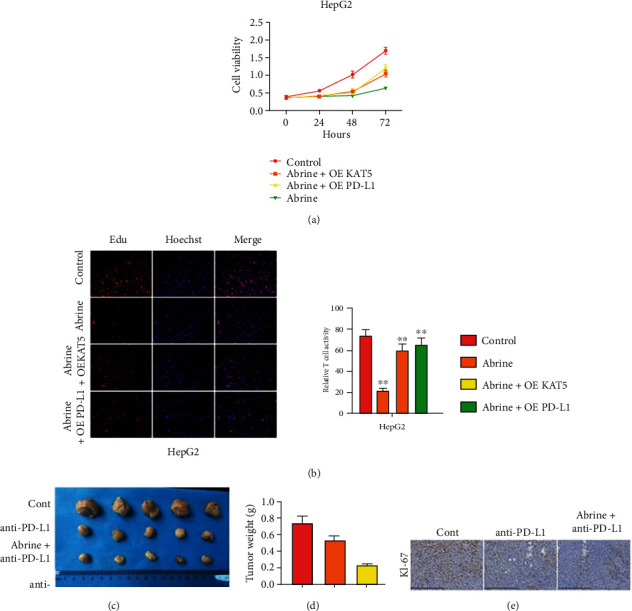
Abrine inhibits liver tumor growth through regulating KAT5/PD-L1 axis. (a, b) HepG2 and Huh7 cells were treated with abrine with or without overexpression of KAT5 or PD-L1. The cell viability (a) and proliferation (b) were measured by CCK-8 and flow cytometry, respectively. (c–e) SCID/nude mice were subcutaneously seeded with HepG2 cells, then treated with PD-L1 antibody and abrine. The tumor size (a) and weight (b) were measured. Level of KI-67 was assessed by IHC staining.

Subsequently, we testified the role of KAT5/PD-L1 axis in liver tumor growth through *in vitro* and *in vivo* experiments. The *in vitro* experiments demonstrated that overexpression of KAT5 and PD-L1 notably recovered abrine-caused suppression of liver cancer cell proliferation (Figures 9(a) and 9(b)). Besides, results from mouse xenograft model indicated that treatment with abrine enhanced the antitumor effect of anti-PD-L1 antibody, manifested by decreased tumor size and weight (Figures 9(c) and 9(d)), as well as the suppressed Ki-67 level (Figure 9(e)). These data suggested that abrine inhibited liver cancer development via regulating KAT5/PD-L1 axis.

## 4. Discussion

Liver cancer, especially the HCC, is becoming the most difficult disease in clinical treatment [23]. Several multikinase inhibitors, such as sorafenib and regorafenib, are currently approved by US Food and Drug Administration for HCC treatment; however, the overall survival of patients is only extended by less than three months with slightly elevated overall response rates [24, 25]. In recent years, cancer immunology has become a highly efficient and widely applied method for cancer therapy [26, 27]. The activation of immune system by blockade of immune checkpoint leads to durable responses during therapy of various cancers [28]. In this work, we disclosed that suppression of PD-L1 by abrine, the extraction from traditional Chinese medicine, exhibited an-cancer effects and activated the T cells through regulating the PD-L1/PD-1 signaling. These findings provided novel evidences of abrine as an anticancer reagent.

As one of the earlies spotted immune checkpoint protein, PD-L1 that expressed by cancer cells and antigen-presenting cells could directly interact with the corresponding PD-1 receptor on T cells and suppresses the cytotoxicity of T cells, which subsequently facilitates cancer cells to escape from immune surveillance [10, 11]. Our data demonstrated that the expression of PD-L1 decreased along with cancer cell death under abrine treatment. It has been widely recognized that stimulation with immune factor IFN-*γ* could elevate the expression of PD-L1 on cancer cell surface, which is regarded as the possible mechanism underlying immune evasion of cancer cells [28]. Noteworthy, we found that abrine could decrease IFN-*γ*-induced PD-L1 in liver cancer cells. Based on the effects of PD-L1 on T cell function, we also evaluated the proliferation and activation of T cells in a coculture system of liver cancer cells and PMSCs. We showed that treatment with abrine significantly decreased the portion of CD4+/PD1+ T cells and CD8+/PD1+ T cells. Abrine treatment facilitated T cell growth and activation.

Increasing studies in some preclinical models have indicated that epigenetic modulators participate in the regulation of immune response, suggesting that targeting epigenetic process is a rationale strategy for cancer therapy [29–31]. Recent studies have found that z-acetylation of KAT5 is associated with transcriptional misregulation of tumor signaling pathways in hepatocellular carcinoma, suggesting that KAT5 may be involved in the pathogenesis of hepatocellular carcinoma [32]. We discovered decreased level of KAT5 in liver cancer cells after abrine treatment. As one of the first acetyltransferase that induce acetylation of both histone and nonhistone proteins, KAT5 has emerged as a promising therapeutic target in cancer treatment [13, 14]. In our study, KAT5 positively regulated the expression of PD-L1. Hence, we studied the epigenetic regulation of KAT5 on PD-L1 and found that KAT5 recruited the H3k27ac and RNA poll II to the promoter region of PD-L1 and promoted gene expression, whereas abrine treatment impaired this recruitment. Moreover, overexpression of KAT5 and PD-L1 abolished the abrine-activated T cell proliferation and activation. Further study with in vitro and in vivo model also manifested that KAT5 participated in abrine-suppressed liver cancer cell growth.

## 5. Conclusion

In conclusion, our study reported that abrine inhibited PD-L1 expression in liver cancer cells via inhibiting epigenetic regulatory function of KAT5, which consequently promoted liver cancer cell proliferation and alleviated cell apoptosis. Moreover, abrine treatment also affected T cell proliferation and activation. Future study on correlation between abrine and KAT5 is still needed.

## Data Availability

The datasets used during the present study are available from the corresponding author upon reasonable request.
